# Key immunity characteristics of diverse stages of brucellosis in rural population from Inner Mongolia, China

**DOI:** 10.1186/s40249-022-00989-7

**Published:** 2022-06-04

**Authors:** Yongzhang Zhu, Li Shi, Yige Zeng, Dongri Piao, Yingbo Xie, Juan Du, Meng Gao, Wei Gao, Junli Tian, Jun Yue, Min Li, XiaoKui Guo, Yufeng Yao, YaoXia Kang

**Affiliations:** 1 School of Global Health, Chinese Center for Tropical Diseases Research, Shanghai Jiao Tong University School of Medicine, Shanghai, China; 2 Department of Immunogenetics, Institute of Medical Biology, Chinese Academy of Medical Sciences & Peking Union Medical College, Kunming, China; 3 Department of Biological Science, Xi’an Jiaotong-Liverpool University, Suzhou, China; 4 State Key Laboratory for Infectious Disease Prevention and Control, Collaborative Innovation Center for Diagnosis and Treatment of Infectious Diseases, National Institute for Communicable Disease Control and Prevention, Chinese Center for Disease Control and Prevention, Beijing, China; 5 Baotou Municipal Center for Disease Control and Prevention, Baotou, Inner Mongolia China; 6 Key Laboratory of Parasite and Vector Biology, Ministry of Health, Shanghai, China; 7 Inner Mongolia Autonomous Region Comprehensive Center for Disease Control and Prevention, Hohhot, Inner Mongolia China

**Keywords:** Human brucellosis, CD4^+^ T cells, CD8^+^ T cells, Th cells, TLR2, TLR4

## Abstract

**Background:**

Brucellosis poses a serious threat to human and animal health, particularly in developing countries such as China. The Inner Mongolia Autonomous Region is one of the most severely brucellosis-endemic provinces in China. Currently, the host immune responses functioning to control *Brucella* infection and development remain poorly understood. The aim of this study is to further clarify the key immunity characteristics of diverse stages of brucellosis in Inner Mongolia.

**Methods:**

We collected a total of 733 blood samples from acute (*n* = 137), chronic (*n* = 316), inapparent (*n* = 35), recovery (*n* = 99), and healthy (*n* = 146) groups from the rural community of Inner Mongolia between 2014 and 2015. The proportions of CD4^+^, CD8^+^, Th1, Th2, and Th17 T cells in peripheral blood and the expression of TLR2 and TLR4 in lymphocytes, monocytes and granulocytes were examined using flow cytometry analysis. The differences among the five groups were compared using one-way ANOVA and the Kruskal–Wallis method, respectively.

**Results:**

Our results revealed that the proportions of CD4^+^ and CD8^+^ T cells were significantly different among the acute, chronic, recovery, and healthy control groups (*P* < 0.05), with lower proportions of CD4^+^ T cells and a higher proportion of CD8^+^ T cells in the acute, chronic, and recovery groups. The proportion of Th1 cells in the acute, chronic, and inapparent groups was higher than that in the healthy and recovery groups; however, there was no significant difference between patients and healthy individuals (*P* > 0.05). The proportion of Th2 lymphocytes was significantly higher in the acute and healthy groups than in the inapparent group (*P* < 0.05). The proportion of Th17 cells in the acute group was significantly higher than that in the healthy control, chronic, and inapparent groups (*P* < 0.05). Finally, the highest expression of TLR4 in lymphocytes, monocytes and granulocytes was observed in the recovery group, and this was followed by the acute, chronic, healthy control, and inapparent groups. There was a significant difference between the recovery group and the other groups, except for the acute group (*P* < 0.05). Moreover, a correlation in TLR4 expression was observed in lymphocytes, monocytes and granulocytes among the five groups (*r* > 0.5), except for the inapparent group between lymphocytes and granulocytes (*r* = 0.34).

**Conclusions:**

Two key factors (CD8^+^ T cells and TLR4) in human immune profiles may closely correlate with the progression of brucellosis. The detailed function of TLR4 in the context of a greater number of cell types or tissues in human or animal brucellosis and in larger samples should be further explored in the future.

**Graphical Abstract:**

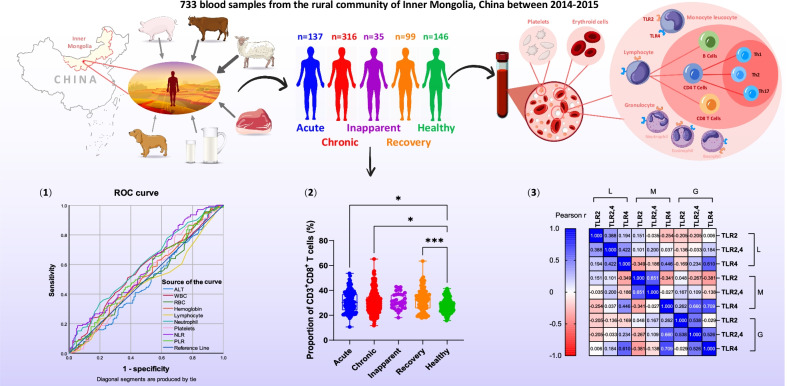

## Background

Brucellosis is a highly contagious bacterial zoonosis that threatens public health worldwide. Brucellosis is caused by bacteria of the genus *Brucella* that is the most important zoonotic pathogen [1]. More than 500,000 human brucellosis cases have been reported annually worldwide [2].

Since 1995, the incidence of human brucellosis has sharply increased in China, and the highest recorded cases occurred in 2014 [1, 3–5]. In 2019, there were still 44,036 cases with an incidence of 3.251/100,000 [1]. Currently, the brucellosis endemic areas have ranged from the northern pastureland areas to the southern coastal and southwestern areas [1, 3]. The Inner Mongolia Autonomous Region is one of the most severely endemic provinces in China and accounted for approximately 40.0% of brucellosis cases in China from 2011 to 2016 [3, 6]. The total seropositivity and incidence rates of human brucellosis among high-risk groups in Inner Mongolia during 2010‒2014 were 35.9% and 18.3%, respectively [7].

The occurrence, disease development, and outcomes of human brucellosis are complex, and thus, the clinical manifestations are also highly diverse. Based on patient epidemiological contact history, clinical manifestations, and laboratory examination results from serological tests and/or positive blood cultures, brucellosis cases have been commonly divided into suspected, clinically diagnosed, definite diagnosed, and inapparent cases. In China, definitively diagnosed cases can be further subdivided into acute, sub-acute, and chronic infections according to the clinical stage of the disease [8, 9]. However, until recently, the timely and accurate diagnoses of human brucellosis and a universally accepted definition for complicated brucellosis has continued to pose a challenge.

*Brucella* is a facultative intracellular gram-negative coccobacillus. Host responses against *Brucella* primarily depend upon cell-mediated immunity provided by CD4^+^ and CD8^+^ T cells [10–12]. As a subset of CD4^+^ T cells, Th1 and Th2 cells also play an important role in brucellosis. Th1 cells and their cytokines (such as IFN-γ and IL-2) act to eliminate bacteria, particularly intracellular bacteria [13–16]. Th2 and its cytokine (IL-4) primarily induce humoral immunity to resist pathogenic infections [17–19]. Th17 cells are different from Th1 and Th2 cells that play a central role in the immune response to intracellular bacteria such as *Brucella* [19, 20]. In addition to adaptive immune responses, the innate immune system plays an important role in *Brucella* infection [21]. Toll-like receptors (TLRs) such as TLR2 and TLR4 located on cytokine-producing cells are involved in *Brucella* recognition by phagocytes to trigger immune responses in different cell types during the early stages of host infection [22].

A few studies have reported the association between peripheral blood T cell subsets or cytokines and patients with brucellosis [18, 19, 23–32]. However, these results are inconsistent. In the current study, we focused on the characteristics of the immune response and investigated the characteristics of T cell subsets (CD4^+^, CD8^+^, Th1, Th2, and Th17) in patients diagnosed with different stages of brucellosis (acute, chronic, inapparent, and recovery) and in healthy individuals from a rural community in Baotou City, Inner Mongolia, China. Moreover, we investigated the expression of TLR2 and TLR4 in lymphocytes, monocytes and granulocytes in this population. Our results will further broaden our understanding of the immune mechanisms that are complicated underlying brucellosis.

## Methods

### Key characteristics of the participants in the five groups

All individuals participating in the current study were long-term inhabitants of two counties (Tuyou and Guyang County in Baotou City) within a rural community in Inner Mongolia Autonomous Region in China, and these participants were primarily engaged in agriculture and animal husbandry work. The questionnaires included socio-demographic, epidemiologic, symptom, and therapy information collected during face-to-face interviews with consenting subjects. The participants with different disease stages, which were initially confirmed according to the criteria of human brucellosis (WS269-2019) issued by the Chinese National Health Commission in 2019 [8], were further divided into the four groups (acute, chronic, inapparent and recovery groups) based on the epidemiological exposure history, clinical presentations, serological tests including rose bengal plate agglutination test (RBPT) and serum agglutination test (SAT), holding time from the initial diagnosis (acute group ≤ 6 months, chronic group > 6 months), and medication history (recovery group with confirmed medication history and negative RBPT/SAT test results). The detailed demographic and diagnostic characteristics of the participants in the four groups and healthy control group are presented in Table 1. Blood samples were obtained from all participants for full blood cell counts and other laboratory examinations.

**Table 1:** Description of the demographic characteristics and diagnosis of brucellosis patients

		Acute (*n* = 137)		Chronic (*n* = 316)		Healthy (*n* = 146)		Inapparent (*n* = 35)		Recovery (*n* = 99)		*P-*value
Subgrouping criteria												
Epidemiologic exposure history		**+**		**+**		** ± **		**+**		**+**		**–**
Symptoms and signs		**+**		**+**		−		**+**		**+**		**–**
Rose Bengal Plate agglutination Test		**+**		**+**		**+**		**+ **		**+**		**–**
Standard agglutination test		**+**		**+**		**+**		**+ **		**+**		**–**
Holding time (Months)		≤ 6		> 6		−		Unknown		Unknown		**–**
Medication history		**+ **		**+ **		**−**		**−**		**+ **		**–**
Demographic features												
Gender (Male/Female)		95/42		240/76		105/41		26/9		77/22		0.519
Age (Years)		50.65 ± 11.36		53.28 ± 9.33		49.38 ± 12.90		46.71 ± 10.37		51.01 ± 10.01		< 0.001
Hematology												
Red blood cells (× 10^12^/L)		4.98 ± 0.59		4.96 ± 0.57		4.92 ± 0.75		5.04 ± 0.51		5.04 ± 0.49		0.567
Hemoglobin (g/L)		146.41 ± 20.60		149.54 ± 16.23		146.58 ± 22.32		150.69 ± 16.39		150.98 ± 15.25		0.157
White blood cells (× 10^9^/L)		5.48 ± 1.46		5.64 ± 1.52		5.82 ± 1.59		5.94 ± 1.46		5.93 ± 1.69		0.127
Lymphocyte (× 10^9^/L)		1.94 ± 0.61		1.87 ± 0.58		1.88 ± 0.60		1.85 ± 0.43		1.97 ± 0.66		0.493
Neutrophil (× 10^9^/L)		2.95 ± 1.38		3.27 ± 1.33		3.45 ± 1.42		3.67 ± 1.30		3.57 ± 1.30		0.001
Platelets (× 10^9^/L)		171.92 ± 67.54		169.96 ± 65.17		172.79 ± 61.00		170.97 ± 55.86		157.38 ± 49.38		0.361
Neutrophil/lymphocyte ratio		1.62 ± 0.91		1.85 ± 0.89		1.98 ± 1.06		2.08 ± 0.97		1.98 ± 1.11		0.009
Platelets/lymphocyte ratio		81.64 ± 25.68		86.84 ± 24.60		84.42 ± 24.60		84.85 ± 18.09		86.37 ± 36.90		0.449
Serum biochemistry												
Alanine transaminase (U/L)		31.28 ± 27.83		28.02 ± 19.05		26.93 ± 16.24		26.74 ± 21.72		32.90 ± 20.88		0.107

+ means have or the test is positive, − means have not or the test is negative

### Reagents

The whole blood cell count and serum alanine aminotransferase (ALT) levels were determined using cytochemical staining-based flow cytometry technology combined with constant temperature (BC-5390CRP, Mindray, China) and radioimmunoassay (Beckman Coulter, AU680, Tokyo, Japan) following the manufacturer’s instructions.

The *Brucella*-specific antibody test was performed using RBPT that has been confirmed by standard tube agglutination test. Diagnostic reagents were kindly supplied by State Key Laboratory for Infectious Disease Prevention and Control, Collaborative Innovation Center for Diagnosis and Treatment of Infectious Diseases, National Institute for Communicable Disease Control and Prevention, Chinese Center for Disease Control and Prevention (Beijing, China).

Anti-human CD8, FITC/IFN-γ, PerCP-Cy5.5/TNF, PE-Cy7/CD3, APC-H7/IL-4, BV421/IL-17A, BV510, TLR2 AlexaFluor647, and TLR4 PE antibodies and a leukocyte activation cocktail with BD GolgiPlug™ stimulants, fixed membrane breaking agents, and RBC lysates were obtained from Becton–Dickinson and Company, USA. T-lymphocyte typing and intracellular cytokine levels were analyzed using a BD LSRII flow cytometer (Becton–Dickinson and Company, USA), and the results were analyzed using BD FACSDiva Software (Version 6.1.2, BD Biosciences) and FCS Express 4 Flow Cytometry (De Novo Software).

### Combination experimental staining of the cell surface and intracellular cytokines in peripheral blood

The CD3^+^ and CD8^+^ cell surface antigens were stained concurrently with the intracellular cytokines IFN-γ, IL-4, IL-17A, and TNF-α in one experimental tube. Anticoagulant peripheral blood (200 μl) and RPMI 1640 (300 μl) were added to a 5 ml BD Falcon tube and stimulated using leukocyte activation cocktail with BD GolgiPlug™ according to the manufacturer’s instructions. After incubation for 4 h in 5.0% CO_2_ at 37 °C, 20 μl CD3 APC-H7 and 20 μl CD8 FITC surface-labeled antibodies were added. Simultaneously, the same type of isotype control tube was prepared and incubated for 20 min in the dark. Next, lysing solution was added to lyse the red blood cells, and the solution was then centrifuged at 300×*g* for 5 min. The cells were re-washed again with staining solution.

After cell-surface staining, the cells were fixed with fixation buffer for 20 min at 4 °C in the dark, and they were then centrifuged and the supernatant was discarded. The cellular suspension was washed twice with permeabilization solution and then intracellularly stained with fluorescently conjugated antibodies (PerCP/Cy5.5 anti-IFN-γ, BV510 anti-IL-17A, PE/Cy7 anti-TNF-α, and BV421 anti-IL-4) and an isotype-matched antibody for 30 min without light. After staining, the samples were washed again, resuspended in permeabilization solution, centrifuged, and the supernatant was discarded. The cells were washed with staining solution, and 500 μl cell washing solution was then added, mixed well, and assessed using a flow cytometer. The results were expressed as the proportion difference compared to the isotypic control.

### TLR2 (CD282^+^) and TLR4 (CD284^+^) on peripheral blood mononuclear cells (PBMC)

Whole blood from all participants cultivated in vitro was stained with AlexaFluor647 labeled anti-human TLR2 and PE-labeled TLR4 antibodies or with isotype controls. Cells were fixed with fixation buffer, and surface expression of TLR2 and TLR4 was analyzed in the lymphocytes, monocytes and granulocytes region using flow cytometry. The results were analyzed as the proportion of positive cells and the mean fluorescence intensity (MFI). The lymphocytes, monocytes and granulocytes populations were defined by gating of the cells according to size (forward scatter). Each cell population was analyzed separately by gating that was achieved using cell size and granularity as parameters. The same software was used to provide data representing the MFI of the respective markers.

### Statistical analysis

Quantitative parameters with normal distribution are presented as mean and standard deviation, and for parameters without normal distribution, median and range values were calculated. The neutrophil/lymphocyte ratio (NLR) and platelet/lymphocyte ratio (PLR) were calculated using these parameters. The receiver operator characteristic (ROC) curve was used to evaluate the predictive value of the hematological parameters including the NLR and PLR. The *χ*^2^ test was used to analyze if there was a sex difference among the five groups. The differences in continuous variables among the five groups were compared using analysis of variance (ordinary one-way ANOVA) (normal distribution) and the Kruskal–Wallis test (non-normal distribution), and Tukey’s and Dunn’s corrections were applied for multiple comparisons. *P* values between 0.01‒0.05, 0.001‒0.01, 0.0001‒0.001, and < 0.0001 were considered statistically significant (*), very significant (**), extremely significant (***), and super significant (****), respectively, and “ns” represents no significance. Correlations were determined using the Spearman’s rank method. The *r* > 0.5 or < − 0.5 with *P* < 0.05 were statistically significant. Statistical analyses were performed using R version 4.0.3 (RStudio, PBC, Boston, MA, USA), SPSS version 21.0 (SPSS, IBM; Inc., Chicago, IL, USA), and GraphPad Prism version 9.0.0 (GraphPad Software, LLC., San Diego, CA, USA).

## Results

### Demographic characteristics and diagnosis of brucellosis patients

A total of 733 blood samples were collected from rural communities in the two counties from 2014 to 2015. The samples were subdivided into five groups based on the diagnostic characteristic results (Table 1), and these groups included the acute (*n* = 137), chronic (*n* = 316), inapparent (*n* = 35), recovery (*n* = 99), and healthy (*n* = 146) groups. Detailed demographic characteristics and clinical and experimental results for the participants are presented in Table 1. There were no significant differences in sex among the five groups (*P* = 0.519); however, there was a significant difference in age among the five groups (*P* < 0.001). Patients in the chronic group were significantly older than those in the healthy and inapparent groups (*P* = 0.023 and 0.001, respectively). For hematological tests, neutrophil count and NLR exhibited significant differences among the five groups (*P* = 0.001 and 0.009, respectively). The neutrophil counts in the healthy and recovery groups were significantly higher than those in the acute group (*P* = 0.019 and 0.007, respectively). The NLR was significantly different among the five groups (*P* = 0.012), where the acute group possessed lower values compared to those of the other groups. However, NLR exhibited no difference after Dunn’s correction in pairwise comparisons (*P* > 0.05). There were no statistical differences in any of the other indices (*P* > 0.05).

### Prediction value of hematological parameters for diagnosis

ROC analysis revealed that certain hematological parameters, including the calculated NLR and PLR, were significantly associated with brucellosis (Table 2). There was a predictive value for ALT between the chronic and recovery groups (*P* = 0.017) and between the inapparent and recovery groups (*P* = 0.027). Platelets were also predictive of differences between the healthy and recovery groups (*P* = 0.010). The neutrophil count was significantly different between the acute group and the chronic, healthy, and inapparent recovery groups (*P* = 0.027, 0.006, 0.024, and 0.006, respectively). NLR was significantly different between the acute group and the chronic, healthy, and inapparent, recovery groups (*P* = 0.006, 0.021, 0.030, and 0.044, respectively). PLR exhibited predictive value only in the acute and chronic groups (*P* = 0.025).

**Table 2:** Progressive significance of ROC curves for hematological parameters and proportions

	Acute-Chronic	Acute-Healthy	Acute-Inapparent	Acute-Recovery	Chronic-Healthy	Chronic-Inapparent	Chronic-Recovery	Healthy-Inapparent	Healthy-Recovery	Inapparent-Recovery
Hematology										
Red blood cells (× 10^12^/L)	0.905	1.000	0.513	0.541	0.915	0.483	0.449	0.527	0.516	0.763
Hemoglobin (g/L)	0.097	0.552	0.402	0.153	0.319	0.992	0.724	0.636	0.306	0.878
White blood cells (× 10^9^/L)	0.457	0.081	0.124	0.169	0.199	0.345	0.399	0.800	0.947	0.816
Lymphocyte (× 10^9^/L)	0.169	0.407	0.276	0.962	0.613	0.924	0.168	0.751	0.450	0.366
Neutrophil (× 10^9^/L)	0.027	0.006	0.024	0.006	0.291	0.324	0.195	0.639	0.785	0.848
Platelets (× 10^9^/L)	0.821	0.576	0.927	0.068	0.412	0.762	0.077	0.828	0.010	0.097
NLR	0.006	0.021	0.030	0.044	0.956	0.448	0.932	0.458	0.818	0.490
PLR	0.025	0.216	0.133	0.509	0.380	0.823	0.197	0.505	0.681	0.329
Serum biochemistry										
Alanine transaminase (U/L)	0.317	0.506	0.221	0.254	0.834	0.275	0.017	0.329	0.062	0.027

*NLR* neutrophil/lymphocyte ratio, *PLR* platelets/lymphocyte ratio

### Percent proportion of CD3^+^CD4^+^ and CD3^+^CD8^+^ T lymphocytes

**Fig. 1:**
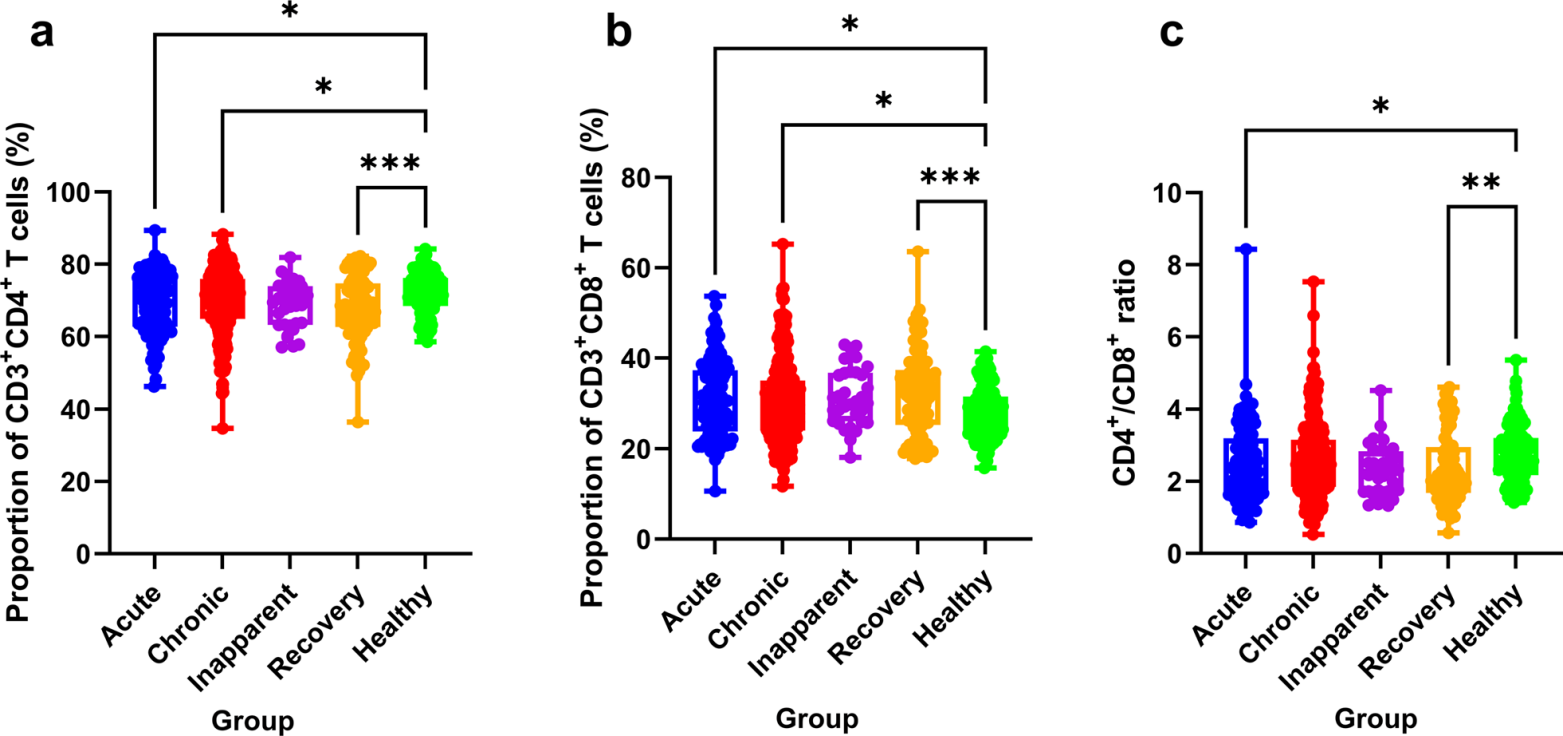
The proportions of CD3^+^CD4^+^ T cells (**a**) and CD3^+^CD8^+^ T cells (**b**) and the CD4^+^/CD8^+^ ratio (**c**) in the peripheral blood from different groups. *P < 0.05; **P < 0.01; ***P < 0.001; ****P < 0.0001

The proportions of CD3^+^CD4^+^ T cells and CD3^+^CD8^+^ T cells in the different groups are provided in Fig. 1a‒c. The proportion of CD3^+^CD4^+^ T cells (Fig. 1a) in the acute, chronic, and recovery groups was significantly lower than that in the healthy group (*P* = 0.024, 0.041, and 0.001, respectively). The proportion of CD3^+^CD8^+^ T cells (Fig. 1b) in the acute, chronic, and recovery groups was significantly higher than that in the healthy group (*P* = 0.023, 0.041, and 0.001, respectively). Additionally, the ratio of CD4^+^/CD8^+^ T cells (Fig. 1c) was significantly higher in the healthy group than in the acute and recovery groups (*P* = 0.050 and 0.003, respectively).

### Proportions of Th1, Th2, and Th17 cells in peripheral blood

**Fig. 2:**
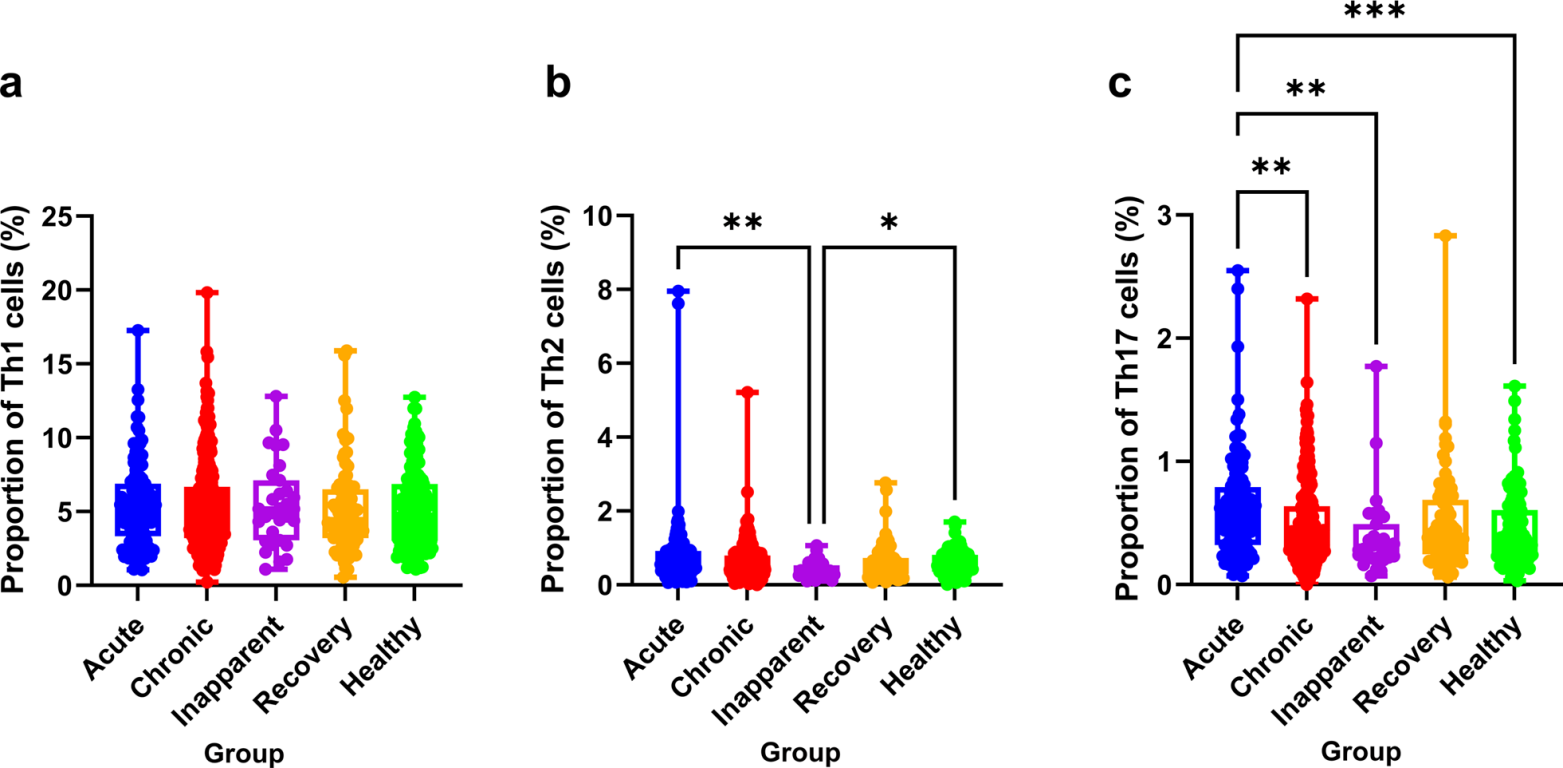
The proportions of Th1 (**a**), Th2 (**b**), and Th-17a (**c**) in the peripheral blood from different groups. *P < 0.05; **P < 0.01; ***P < 0.001; ****P < 0.0001

The proportions of Th1, Th2, and Th17a in the different groups are presented in Fig. 2a‒c. For Th1 (CD4^+^ IFN-γ) (Fig. 2c), the proportion of Th1 lymphocyte expression in the PBMCs in the acute (5.415 ± 2.773), chronic (5.315 ± 2.992), and inapparent (5.460 ± 2.822) groups was higher than that in the healthy (5.148 ± 2.671) and recovery (5.090 ± 2.873) groups. However, there was no significant difference between the groups (*P* > 0.05). For Th2 (CD4^+^ IL-4) (Fig. 2b), the proportion of Th2 lymphocytes in the acute and healthy groups was significantly higher than that in the inapparent group (*P* = 0.003 and 0.045, respectively). Although the proportion in the acute group (0.748 ± 0.870) was higher than that in the healthy group (0.590 ± 0.313), there were no significant differences between the two groups (*P* > 0.05). For Th17 (CD4^+^ IL-17A) (Fig. 2c), the proportion of Th17 lymphocytes in the acute group was significantly higher than that in the chronic, healthy, and inapparent groups (*P* = 0.003, < 0.001, and 0.003, respectively). Additionally, the inapparent group (0.275 ± 0.518) was also lower compared to the other four groups (*P* > 0.05).

### Expression of TLR2 and TLR4 in lymphocytes, monocytes and granulocytes

**Fig. 3:**
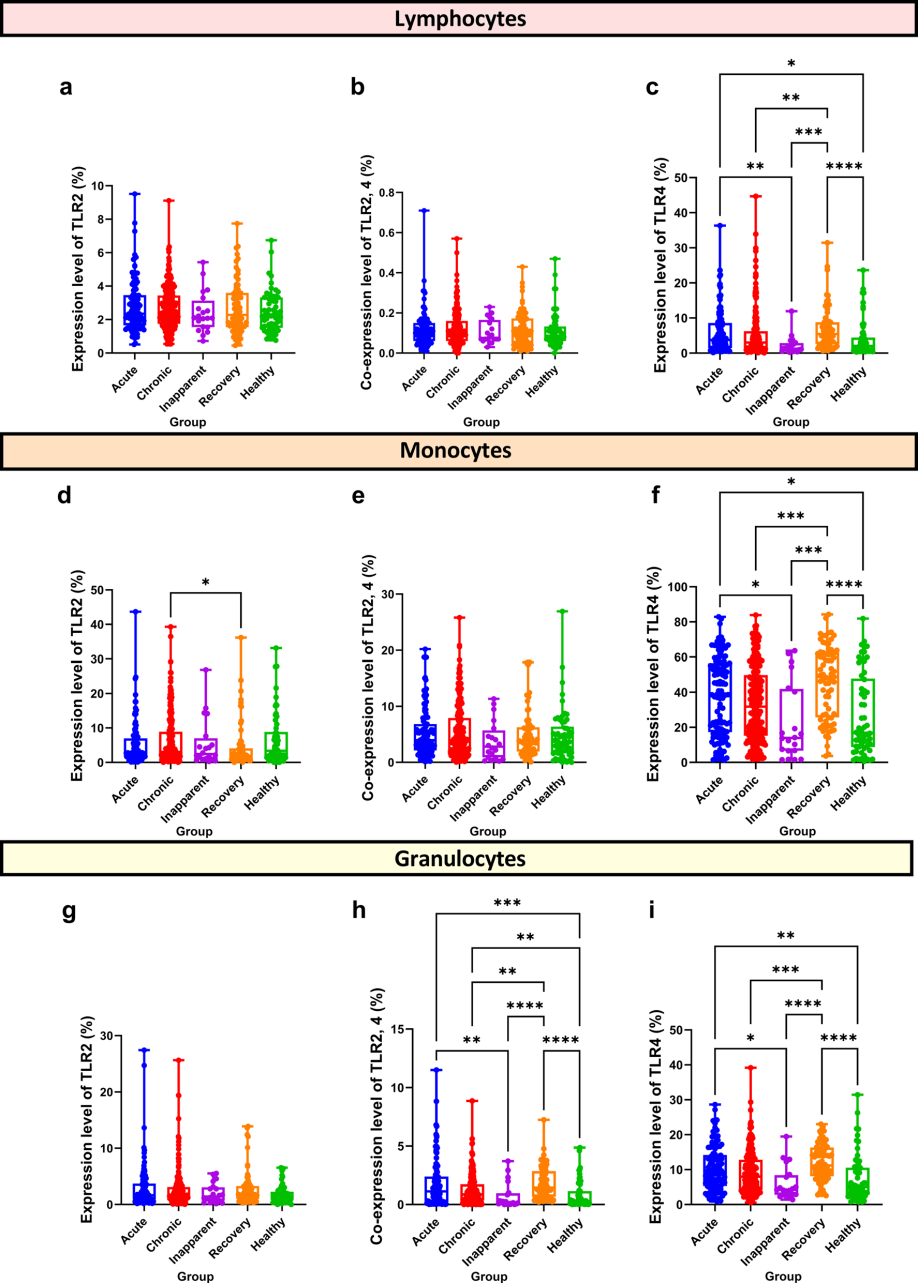
Comparison of the levels of TLR2 and TLR4 and the co-expression of TLR2,4 in lymphocytes (**a**‒**c**), monocytes (**d**‒**f**), and granulocyte (**g**‒**i**) among the five groups of participants. *P < 0.05; **P < 0.01; ***P < 0.001; ****P < 0.0001

The blood samples from all participants exhibited the presence of effective TLR expression in lymphocytes, monocytes and granulocytes. The levels of TLR2 and TLR4 and the co-expression of TLR2,4 in lymphocytes, monocytes and granulocytes are presented in Fig. 3a‒i.

There was no significant difference in the expression level of TLR2 or the co-expression of TLR2,4 in the different groups of lymphocytes (Fig. 3a, b). The expression levels of TLR4 in lymphocytes (Fig. 3c) in the acute group were higher than those in the healthy and inapparent groups (*P* = 0.014 and 0.010, respectively). TLR4 expression levels in the recovery group were also higher than those in the chronic, healthy, and inapparent groups (*P* = 0.008, < 0.001, and < 0.001, respectively).

In monocytes, the expression of TLR2 (Fig. 3d) in the chronic group was higher than that in the recovery group (*P* = 0.014). The expression of TLR2 and TLR4 was not significantly different among the five groups (Fig. 3e). The expression levels of TLR4 (Fig. 3f) in the chronic, healthy, and inapparent groups were significantly lower than those in the recovery group (*P* < 0.001, < 0.001, and < 0.001, respectively). The expression of TLR4 in the acute group was also elevated compared to that in the healthy and inapparent groups (*P* = 0.015 and 0.271, respectively).

The expression of TLR2 in granulocytes (Fig. 3g) was not significantly different among the five groups. The co-expression of TLR2,4 and the individual expression of TLR4 in granulocytes from the acute and recovery groups exhibited the same expression tendency, and the levels were higher than those of the healthy group (Fig. 3h, i) and exhibited significant differences (*P* < 0.001 and < 0.001 for co-expression of TLR2,4; *P* = 0.005 and < 0.001, respectively, for expression of TLR4). Significant differences were also observed between the recovery group and the chronic and inapparent groups (*P* = 0.002 and < 0.001 for co-expression of TLR2,4; *P* < 0.001 and < 0.001, respectively, for expression of TLR4). Both expression levels **(**Fig. 3h, i) in the acute group were higher than those in the inapparent group (*P* = 0.009 and 0.015, respectively). Additionally, the co-expression of TLR2,4 in granulocytes (Fig. 3h) was higher in the chronic group than in the healthy group (*P* = 0.010).

### Expression correlation of TLR2 and TLR4 in lymphocytes, monocytes and granulocytes

**Fig. 4:**
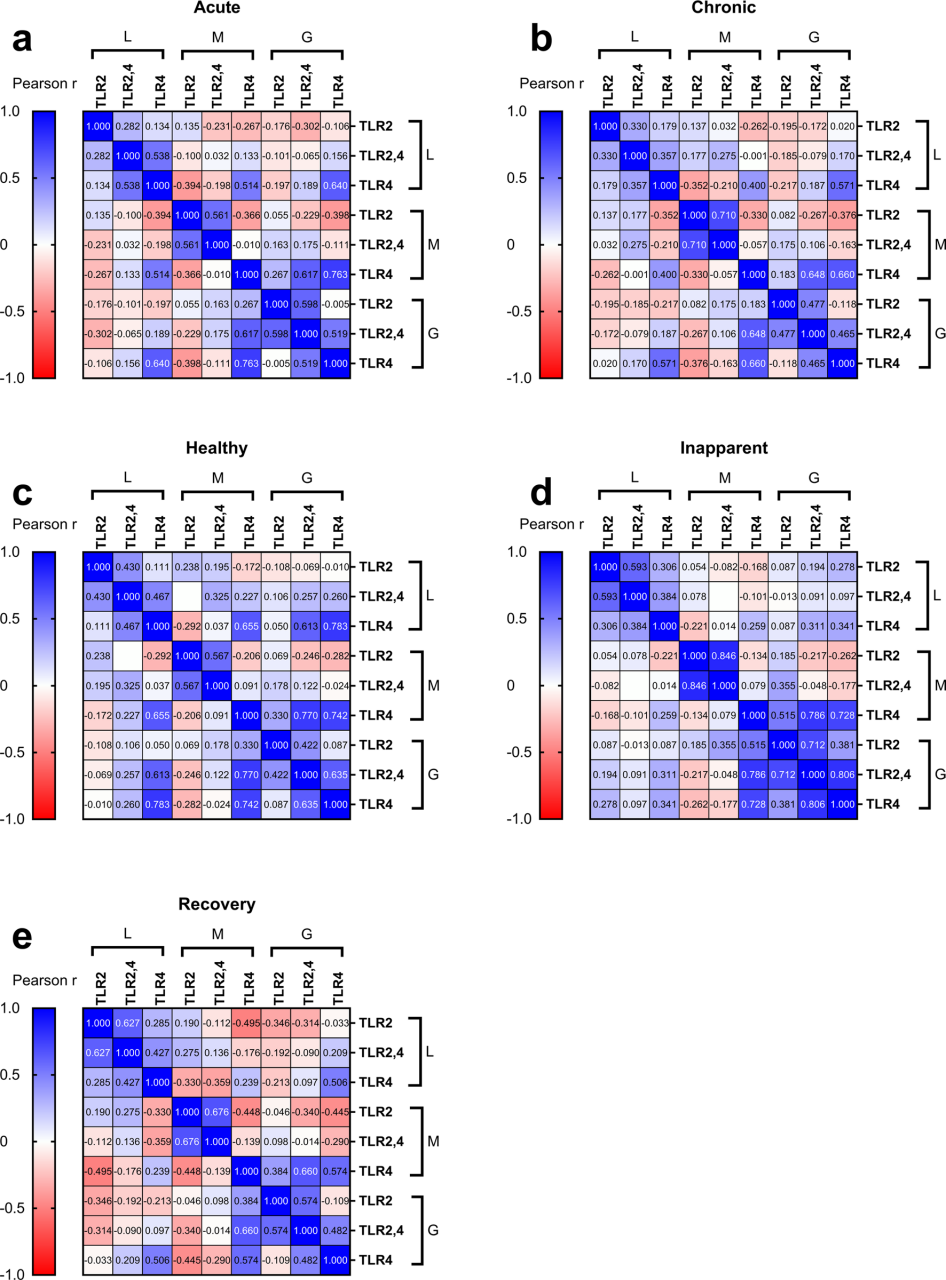
Heatmaps with Pearson correlation coefficient. The expression of TLR2 and TLR4 in the lymphocytes, monocytes and granulocytes among the acute (**a**), chronic (**b**), healthy (**c**), inapparent (**d**), and recovery (**e**) groups. The color density represents the magnitude of the correlations, with blue color indicating positive and red color indicating negative. *L* lymphocytes, *M* monocytes, *G* granulocytes

The correlation between TLR2 and TLR4 expression in lymphocytes, monocytes and granulocytes is presented in Fig. 4a‒e. The expression level of TLR4 in lymphocytes, monocytes and granulocytes was correlated with that in the acute and healthy groups, and the Pearson r was 0.514 and 0.655 (*P* < 0.001 and < 0.001, respectively). Except for the inapparent group, the expression levels of TLR4 in lymphocytes and granulocytes were correlated in the other four groups, with Pearson r values of 0.640 in the acute group, 0.571 in the chronic group, 0.783 in the healthy group, and 0.506 in the recovery group (*P* < 0.001, < 0.001, < 0.001, and < 0.001, respectively). The TLR expression levels of granulocytes, monocytes were correlated in all the five groups, and Pearson r was 0.763 in the acute group, 0.660 in the chronic group, 0.742 in the healthy group, 0.728 in the inapparent group, and 0.574 in the recovery group (*P* < 0.001, < 0.001, < 0.001, < 0.001, and < 0.001, respectively). Additionally, the co-expression levels of TLR2,4 in granulocytes were correlated with the expression levels of TLR4 in monocytes, with Pearson r values of 0.671, 0.648, 0.770, 0.786, and 0.660 in the acute, chronic, healthy, inapparent, and recovery groups, respectively (*P* < 0.001, < 0.001, < 0.001, < 0.001, and < 0.001, respectively). In the healthy group (Fig. 4c), co-expression levels of TLR2,4 in granulocytes were also associated with TLR4 expression levels in lymphocytes (*r* = 0.613, *P* < 0.001).

Additionally, the expression levels of TLR2 and TLR4 and the co-expression levels of TLR2,4 were correlated in the same type of cells. For lymphocytes, the expression of TLR4 in the acute group (Fig. 4a) was correlated with the co-expression of TLR2,4 (r = 0.538, *P* < 0.001), while the expression of TLR2 in the inapparent (Fig. 4d) and recovery groups (Fig. 4e) was correlated with the co-expression of TLR2,4 (*r* = 0.593, *r* = 0.627; *P* < 0.001 and *P* < 0.001, respectively).

In monocytes, the expression level of TLR2 was correlated with the co-expression level of TLR2,4 in four of the five groups (Fig. 4a–e), where the Pearson r was 0.561 in the acute group, 0.710 in the chronic group, 0.567 in the healthy group, 0.846 in the inapparent group, and 0.676 in the recovery group (*P* < 0.001, < 0.001, < 0.001, < 0.001, and < 0.001, respectively). Additionally, the expression levels of TLR2 and TLR4 in monocytes were correlated (*r* = 0.515, *P* < 0.001).

In granulocytes, the co-expression levels of TLR2,4 from the acute (Fig. 4a), inapparent (Fig. 4d), and recovery (Fig. 4e) groups were all related to the expression levels of TLR2, and Pearson r values were 0.598, 0.712, and 0.574, respectively (*P* < 0.001, < 0.001, and < 0.001, respectively). The co-expression level of TLR2,4 was also correlated with the expression level of TLR4 in the acute group (*r* = 0.519, *P* < 0.001), the healthy group (*r* = 0.635, *P* < 0.001), and the inapparent group (*r* = 0.806, *P* < 0.001).

## Discussion

The host immune response plays a crucial role in host defense against intracellular bacteria such as *Brucella* species. In the current study, we investigated the characteristics of T cell subsets (CD4^+^, CD8^+^, Th1, Th2, and Th17) and TLR2 and TLR4 in lymphocytes, monocytes and granulocytes in acute, chronic, inapparent, recovery, and healthy groups in a Chinese Han population.

Olt et al. reported that lower NLR values were significantly associated with brucellosis by ROC analyses [33]. In the current study, we determined that NLR values were significantly associated with the acute group that exhibited lower NLR values. One of the reasons that the acute group exhibited a lower NLR could be due to the lower neutrophil count in the acute group. These results indicated that low neutrophil count and NLR could distinguish the acute group from the other groups.

Cell-mediated immunity plays an important role in protecting humans against *Brucella* infection [10–12]. During cell-mediated immunity, antigen-presenting cells and T lymphocytes help the host resist or clear *Brucella* infection [10, 34]. In 2018, Zheng et al. reported a significantly decreased proportion of CD4^+^ T cells and an increased proportion of CD8^+^ T cells in human brucellosis patients compared to levels in healthy subjects [30]. In the current study, we also observed that the proportion of CD4^+^ T cells in the acute, chronic, and recovery groups was significantly lower than that in the healthy group. Moreover, the proportion of CD8^+^ T cells was significantly higher in the acute, chronic, and recovery groups than in the healthy group. In 2005, Akbulut et al. reported that CD4^+^ T cell levels were significantly lower in brucellosis patients than in the healthy group [24]. For CD8^+^ T cells, Moreno-Lafont and Skendros et al. reported an increase in cytotoxic CD8^+^ T cell disturbances in T-cell immunity in chronic persistent/relapsing brucellosis patients [31, 35]. These results confirmed that CD8^+^ T cells are more important than CD4^+^ T cells in controlling *Brucella* infection [36].

In 2018, Zheng et al. reported that the proportion of Th1 cells was increased in human brucellosis patients; however, there was no significant difference between the patients and the controls [30]. Xu et al. reported that the frequency of Th1 cells was significantly higher in patients with brucellosis than in healthy controls [28]. In the current study, we observed that the proportion of Th1 cells in the acute, chronic, and inapparent brucellosis groups was higher than that in the healthy and recovery groups; however, neither exhibited significantly differences between patients and control individuals. Th1 cells primarily mediate cellular immunity and control bacterial infection by secreting IFN-γ and IL-2 that function to destroy bacterial infection, particularly regarding intracellular bacteria such as *Brucella* species in macrophages [13–16]. In 2019, Xu et al. determined that the mean serum levels of IFN-γ were significantly higher in patients with brucellosis compared to those in healthy individuals [28]. Lin et al. also observed that serum IFN-γ levels were higher in the patient group than those in the healthy group [18]. These results indicate that Th1 cells and their secreted cytokines play important roles in controlling *Brucella* infection.

The proportion of Th17 cells in patients with acute brucellosis increases significantly and subsequently decreases after treatment [37]. In the current study, we observed that the proportion of Th17 cells in the acute group was significantly higher than in the chronic, healthy, and inapparent groups. In 2019, Zheng et al. reported that the proportion of Th17 cells in acute and chronic patients was higher than that in healthy control. Additionally, they also determined that the levels of effector molecules in Th17 cells (IL-17A and IL-17F) in the acute and chronic groups were higher than those in the healthy control group. In 2020, Lin et al. reported that serum IL-17 levels were higher in brucellosis patients than in controls (*P* < 0.05) [18]. Moreover, they also observed that IL-17 levels in the serum were significantly higher in the acute group than in the chronic group [18]. These results indicated that Th17 cells and their effector molecules such as those active during the acute stages of *Brucella* infection could participate in cellular immunity against *Brucella* infection.

In addition to adaptive immune responses, the innate immune system plays an important role in *Brucella* infection [21]. TLRs play central roles in the induction of innate immune responses and also in the subsequent development of adaptive immune responses [12]. Our results revealed that the highest expressions of TLR4 in lymphocytes, monocytes and granulocytes were in the recovery group, and then followed by the acute, chronic, healthy, and inapparent groups. Additionally, TLR4 expression in one cell type was positively correlated with that in the other cell types. For example, the high expression of TLR4 in lymphocytes was positively correlated with that in monocytes and granulocytes. These interesting findings were almost identical expression patterns of TLR4 observed in lymphocytes, monocytes and granulocytes, indicating that TLR4 expression could play a vital role in the resistance to *Brucella* infection in humans. In 2004, Campos et al. demonstrated the role of TLR4 in triggering an immune response against *Brucella*, thus identifying TRL4 as an important molecule in the control of brucellosis [38]. Copin et al. reported that TLR4 and not TLR2 significantly decreased resistance to *B. melitensis* infection in an experimental model [39]. Pei et al. demonstrated that TLR4 is also important for effective *B. abortus* internalization by macrophages [40]. However, Weiss et al. surprisingly observed that TLR4 is not required for the clearance of *Brucella *in vivo [41]. Moreover, Barquero-Calvo et al. demonstrated that the replication of *B. abortus* S19 and S2308 in vivo occurs independently of TLR4 [42]. The reasons for these discrepancies are currently unknown; however, they may be partially due to the different roles of TLR4 in different host cells or to the different *Brucella* strains that were used. Additionally, Pei et al. reported that TLR2 and TLR4 contributed little to the control of *Brucella* spleen infection; however, a significant contribution to the clearance of lung infection was described, thus suggesting that the potential roles of TLRs may be related to host tissue specificity [43].

Several studies have reported that TLR2 plays no role in controlling *B. abortus* infection in vivo [38, 41]. In the current study, we determined that TLR2 expression was not significantly different in lymphocytes, monocytes and granulocytes among the five groups, except for the chronic and recovery groups in monocytes. These results further indicate that TLR2 may not play a role in controlling *B. abortus* infection.

Finally, there are two main limitations in the current study. Firstly, it is necessary to include the levels of cytokines in the peripheral serum in the current association analysis, which could display the association between T cells, its secreted cytokines and brucellosis. Unfortunately, we are unable to test the levels of cytokines now, which could be one main limitation in the current study. In addition, our primary purpose focused on the immunity characteristics of diverse stages of brucellosis in rural population, thus, the individuals’ chronic disease conditions, like diabetes, hypertension, and autoimmune diseases, were not comprehensively investigated, which would be another limitation within this study.

## Conclusions

In the current study, we comprehensively investigated the detailed roles of TLR2 and TLR4 in different cells at different stages of *Brucella* infection, including the acute, chronic, inapparent, and recovery stages. Human immune response profiles such as CD4^+^ T, CD8^+^ T cells, Th cells, and TLR profiles could correlate with disease susceptibility or protection. Especially, two key factors (CD8^+^ T cells and TLR4) in human immune profiles could be markedly associated with the progression of brucellosis. In the future, the detailed function of TLR4 should be further explored using a more significant number of human cell types or tissues and in larger sample populations.

## Data Availability

Not applicable.
